# Common features in spatial livestock disease transmission parameters

**DOI:** 10.1038/s41598-023-30230-w

**Published:** 2023-03-02

**Authors:** Gert Jan Boender, Thomas J. Hagenaars

**Affiliations:** Wageningen Bioveterinary Research, P.O. Box 65, 8200 AB Lelystad, The Netherlands

**Keywords:** Infectious diseases, Computational models, Power law

## Abstract

The risk of epidemic spread of diseases in livestock poses a threat to animal and often also human health. Important for the assessment of the effect of control measures is a statistical model quantification of between-farm transmission during epidemics. In particular, quantification of the between-farm transmission kernel has proven its importance for a range of different diseases in livestock. In this paper we explore if a comparison of the different transmission kernels yields further insight. Our comparison identifies common features that connect across the different pathogen-host combinations analyzed. We conjecture that these features are universal and thereby provide generic insights. Comparison of the shape of the spatial transmission kernel suggests that, in absence of animal movement bans, the distance dependence of transmission has a universal shape analogous to Lévy-walk model descriptions of human movement patterns. Also, our analysis suggests that interventions such as movement bans and zoning, through their impact on these movement patterns, change the shape of the kernel in a universal fashion. We discuss how the generic insights suggested can be of practical use for assessing risks of spread and optimizing control measures, in particular when outbreak data is scarce.

## Introduction

Epidemics of highly contagious diseases in livestock such as Foot and-Mouth Disease (FMD) and high pathogenic avian influenza (HPAI) can have tremendous socio-economic consequences as well as devastating effects on animal health^1,2^. For developing effective contingency planning for the control of such epidemics, it is essential to use the data from previous epidemics to gain as much insight as possible in the quantitative characteristics of transmission, in particular of between-farm transmission^3^. These characteristics may include the observed effect of control measures such as animal movement standstill or zoning^4^. By means of using mathematical models fitted to describe these characteristics, past or current outbreaks in a given country are often being used to make extrapolations to current or future transmission risks in that same country^5,6^. For countries with no earlier or no informative outbreak, one may resort to extrapolation from epidemic patterns observed in other countries, addressing uncertainties about model representativity for the country of interest e.g. by exploring the sensitivity of the model outcomes to possible differences in parameter values^7^. For emerging diseases for which there is no previous epidemic that can be analyzed, what can we learn from the patterns observed in epidemic data of other livestock diseases? Here we present a general framework for the spatial transmission of livestock diseases that can help to underpin model extrapolations between control strategies, between countries, and also between diseases. It is built on the comparison of epidemic transmission risk patterns across a range of livestock diseases.

Our framework uses the transmission kernel as a central element in the modelling approach. Transmission kernels describe the distance-dependent probability of transmission from an infected to a susceptible farm, and have been used to describe the between farm transmission of different animal diseases^5,6^. Use of a transmission kernel avoids the modelling of specific transmission pathways where these are poorly known, and allows both the construction of risk maps as well as model simulation studies of the effectiveness of control measures^5,6,8,9^. The transmission kernel also facilitates the comparison of the distance-dependent characteristics of transmission between epidemics, between diseases, and between phases in one and the same epidemic differing in applied control measures, in particular in the type of animal movement restrictions that were applied^10–12^.

For the interpretation of the comparative kernel analyses results we can build on a body of literature that uses spatial kernels to describe movement and dispersal patterns^13,14^. One of the elements from this literature is a distinction between thin-tailed (i.e. exponentially bounded) and fat-tailed (i.e. power-law) kernels^15^. Whereas thin-tailed kernels generate ‘diffusive’ dispersal patterns with constant-speed travelling waves, a fat-tailed kernel produces ‘super-diffusive’ behavior lacking a finite velocity and yielding a patchy dispersal pattern^15–18^. In the description of animal movement, thin-tailed kernels are a signature for an underlying Brownian random walk and fat-tailed kernels for Lévy-walk patterns; based on this distinction several studies demonstrate Lévy-walk patterns for animal movement^19–22^. Also human mobility appears to follow a Lévy walk^23,24^, and this could be of relevance to between-farm disease transmission as transmission between farms is likely caused in part by humans moving between the farms and thereby acting as passive vectors.

An important subtlety described by the so-called ‘truncated’ Lévy-walk model is the phenomenon that the fat-tailed/power-law behaviour is truncated by an exponential decline setting in above a cut-off distance scale. This truncated version of the Lévy-walk model describes super-diffusive movement on a distance small compared to, and diffusive movement on a scale large compared to, the cut-off distance^25^. In the case of human mobility, such a cut-off distance scale may reflect the existence of an area within which most of the movements are confined, e.g. an urban area for the movements of commuters^26^. For human travel mobility patterns the power-law exponent in the truncated Lévy walk appears to have a universal value of about 1.6^17,23^.

The aim of this article is to yield insight into the similarities and differences between a range of spatial patterns of animal disease transmission by comparison of the corresponding transmission kernels. In particular, we seek to investigate to which extent the observed between-farm transmission patterns can be categorized and interpreted using insights from the study of movement and dispersal patterns. We will use one and the same kernel parametrisation throughout such that parameter values can be directly compared. In our previous kernel analyses of between-farm transmission we have mostly adopted a so-called Cauchy form (defined below) to parameterize the kernel, motivated in part by the fact that it was identified as having the lowest AIC amongst a set of alternatives studied in^6^, in which the Lévy-walk kernel was not included. Recent analyses suggest that the (non-truncated) Lévy-walk kernel produces an even better fit to spatial transmission data from certain livestock disease epidemics^10,27^.

Therefore we re-analyzed eight epidemic datasets available to us using the Lévy-walk kernel; basic characteristics of these eight datasets are listed in Table 1 and 2. In addition to this, we assembled from the literature three Lévy-walk kernels estimated for other animal disease epidemic datasets. Together, the 11 datasets comprise a broad set of different host–pathogen combinations, including viral and bacterial pathogens. The resulting set of 11 Lévy-walk kernels, each one determined by estimated values for four kernel parameters, allows us to compare the results to see if similarities extend across a broad set of livestock disease transmission patterns. Two of the four parameters are of highest importance in our analysis, as together these two largely determine the shape of the distance dependence. As a formal framework for the comparison between the 11 estimated kernels, we (1) apply a hierarchical clustering analysis to the set of 11 pairs of maximum-likelihood values for these two kernel shape parameters, and (2) correlate the clusters found to the type of control strategies applied during the 11 epidemics. A descriptive overview of the epidemics analyzed is given as part of the Materials and Methods. In the Results, the estimated Lévy-walk model parameters for each epidemic are presented, the hierarchical clustering analysis is carried out, and clusters found are correlated with applied control strategies. In the Discussion, we present the insights obtained and their practical relevance for assessing risks of spread and optimizing control measures, in particular when outbreak data is scarce.

**Table 1:** List of 11 epidemic datasets analyzed, ordered by applied control strategy (in order of increasingly stringent measures: no movement ban nor zoning (NMNZ), no movement ban but zoning (NMZ), and a movement ban (M)) and year. Datasets 1 and 7 represent two time periods of one and the same epidemic; for brevity we often refer to all 11 datasets as ‘epidemics’ in the main text.

Nr	Disease	Year	Country	Strategy	Kernel model of existing analysis
1.	FMD^10^	Before 23rd February 2001	UK	NMNZ	Lévy Walk
2.	SVD^12^	2006	Italy	NMNZ	Reference
3.	Q fever^28^	2007–2010	The Netherlands	NMNZ	Reference
4.	BT^11^	2006	Belgium	NMZ	Reference
5.	BT^11^	2006	Germany	NMZ	Reference
6.	CSF^29^	1997–1998	Netherlands	M	Reference
7.	FMD^10^	Post 23rd February 2001	UK	M	Lévy Walk
8.	FMD^8^	2001	The Netherlands	M	Reference
9.	HPAI^6^	2003	The Netherlands	M	Reference
10.	SVD^12^	2007	Italy	M	Reference
11.	FMD^27^	2010	Japan	M	Lévy Walk

**Table 2:** Basic characteristics of eight epidemics/epidemic datasets analyzed. For further details we refer the reader to the original publications on these datasets.

Nr	Disease	Disease type	Affected species	Population size	Number of outbreaks	Reference
2^12^	SVD	viral	Pigs	7600	36	^12,40^
3^28^	Q fever	bacterial zoonotic	Goats	404	176	^28^
4^11^	BT	vector-borne viral	Cattle Sheep	239,336	1119	^11,41,42^
5^11^	BT	vector-borne viral	Cattle Sheep	87,007	880	^11,43^
6^29^	CSF	viral	Pigs	23,131	428	^29^
8^8^	FMD	viral	Cattle Goats	94,506	26	^8,44^
9^6^	HPAI	viral zoonotic	Poultry	5360	241	^6^
10^12^	SVD	viral	Pigs	7600	17	^12,40^

## Material and methods

We assemble and compare the Lévy-walk kernel fits to 11 different between-farm transmission datasets. These datasets are listed in Table 1. Three of the datasets were already analyzed using Lévy-walk kernels in the literature, namely two parts of the UK 2001 FMD epidemics and the 2010 FMD epidemic in Japan^10,27^. The remaining eight datasets of livestock epidemics or parts of epidemics^6,8,11,12,28,29^ are reanalyzed here by fitting truncated Lévy-walk kernels. These eight comprise the 2006 Q fever epidemics in the Netherlands, two parts of the Swine Vesicular Disease (SVD) epidemics in 2006/2007 in Italy, the 2001 FMD epidemic in the Netherlands, the 2003 HPAI epidemic in the Netherlands, the 1997/1998 Classical Swine Fever (CSF) epidemic in the Netherlands, and the 2006 Blue Tongue (BT) epidemics in Germany and Belgium. These eight epidemic datasets were previously analyzed individually using the ‘Cauchy’ form of the transmission kernel

$$ \lambda \left( r \right) = \frac{{\lambda_{0} }}{{1 + \left( {\tfrac{r}{{r_{0} }}} \right)^{\alpha } }}, $$

in which $r$ is the Euclidean distance between an infectious and a susceptible farm, ${\lambda }_{0}$ represents the amplitude of the transmission kernel and is interpreted as the transmission hazard for very small distance (‘distance zero’) between the infectious and the susceptible farm, and ${r}_{0}$ is a characteristic distance, also referred to as ‘kernel offset’^10^. It is the distance where the transmission hazard has become half as large as at distance zero, and therefore it has an influence on the distance dependence of the kernel for distances up until a few times ${r}_{0}$. The parameter $\alpha$ is a scaling exponent that determines how fast the long-distance transmission probability declines, and its influence on the kernel shape dominates over the influence of ${r}_{0}$ for distances a few times larger than ${r}_{0}$ and beyond. In more detail, the role of $\alpha$ is that its value indicates whether transmission is short-ranged ($\alpha >3$), intermediate-ranged ($2<\alpha \le 3$), or long-ranged ($\alpha \le 2$)^11^. In this paper we keep the Cauchy kernel results for reference, to compare the fits to those using the Lévy-walk kernel. We use the following parametrization for the (truncated) Lévy-walk kernel^23^:

$$ \lambda \left( r \right) = \frac{{\lambda_{0} }}{{\left( {1 + \tfrac{r}{{r_{0} }}} \right)^{\alpha } }}\exp \left( { - \frac{r}{\kappa }} \right), $$

in which the additional parameter $\kappa$ is a cutoff distance truncating the Lévy-walk behavior to a finite spatial range. The parameter estimates are obtained Maximum Likelihood (ML) estimation, and confidence bounds using the likelihood-ratio test. As the kernel amplitude λ_0_ does not inform about the distance dependence of transmission, we only report its estimated values in the Supplementary Information for completeness. We note that the estimate for the cutoff distance $\kappa$ is only informative if it is smaller than the ‘extent’ of the area spanned by the dataset; if larger, then no truncation is detected on the distance scales covered by the data. The likelihood that is maximized for the kernel estimation is constructed in the following way. For each farm an infection status (susceptible (S), becoming infected (C), latent (E), infectious (I), removed (R)) is assigned per time unit (day or week depending on dataset). Based on this, we obtain a set of possible infection events (a farm $i$ with status I and a farm $j$ with status C in the same time unit) and a set of escape events (a farm $i$ with status I and a farm $j$ with status S in the same time unit). ${\Lambda }_{j}^{\text{C}}$ is the set of all farms $i$ with status I at the start of the time unit in which farm $j$ has status C. ${\Lambda }_{j}^{\text{S}}$ is set of all farms $i$ with status I for at least one time unit during which farm $j$ has status S and ${N}_{ij}^{\text{S}}$ is the number of time units for which $i$ has status I and $j$ has status S. As the escape probability during each one of such time units is given by ${\text{exp}}\left(-\lambda \left({r}_{ij}\right)\right)$, where ${r}_{ij}$ is the Euclidean distance between farm $i$ and farm $j$, it is easily derived that the combination of all events has the following likelihood:

$$L\left({\lambda }_{0},\alpha ,{r}_{0},\kappa \right)=\prod_{j\in\Phi }\left(1-\prod_{i\in {\Lambda }_{j}^{\text{C}}}{\text{exp}}\left(-\lambda \left({r}_{ij}\right)\right)\right) \prod_{k\in\Omega }\prod_{i\in {\Lambda }_{k}^{\text{S}}}{\text{exp}}\left(-\lambda \left({r}_{ik}\right){N}_{ik}^{\text{S}}\right),$$

where $\Phi$ is the set of all case farms except for the index case farm, and $\Omega$ the set of all farms except for the index case farm.

The kernel is defined as a transmission hazard between two given individual farms, and its parameters are assumed to be homogeneous across the geographical area where the outbreaks occurred; in particular these parameters are assumed to be independent of farm density. To our knowledge there are no examples in the literature of epidemics in livestock where a regionally stratified kernel analysis produces significant differences in the shape parameters of the transmission kernel; e.g.^30^ noted that for FMD in 2001 in Great Britain ‘No significant regional [….] differences in the spatial transmission kernel were found, although statistical power was limited in areas outside Cumbria by lower case incidence.’

During the 11 epidemics the control strategy was either a movement ban (M), or no movement ban but zoning (NMZ), or no movement ban nor zoning (NMNZ). Zoning was applied in the 2006 BT epidemic in Germany with a typical zone radius of about 20 km and in the 2006 BT epidemic in Belgium, with spillover to the Netherlands and France, in which during the main part of the epidemics Belgium was considered to be one single zone corresponding approximately to a radius of about 140 km (half the extent of the country)^11,31^. Using the Akaike’s Information Criterion (AIC) the model fit of the Lévy-walk kernel was compared that of the reference (i.e. Cauchy) kernel (AIC_0_). Differences in AIC’s are considered to be significant if larger than 2^32,33^.

In order to objectively identify categories of epidemic transmission patterns we carried out a hierarchical clustering analysis using the estimated parameter pairs $\left\{\alpha ,\kappa \right\}$^34^. Here we used a Euclidean distance function after rescaling both parameters to take values between 0 and 1. The rescaling was carried out using a logistic scaling function, in which for each of the two parameters the scaling factor was set to the median value across all 11 estimates. To assess the statistical significance of correlation between the clusters found and the type of control strategies applied during the 11 epidemics, we calculate a p-value by basic combinatorics.

## Results

The estimated parameters for the Lévy-walk kernel for the 11 epidemics are given in Table 3; for plots of the 11 kernel shapes we refer to Fig. S1. Amongst the eight epidemics we re-analyzed here, only epidemics 4 and 5 correspond to a finite spatial range $\kappa$. For epidemics 2, 3, 6 and 8–10, the estimation yielded values for the cutoff parameter $\kappa$ that were much larger than the spatial extent of the datasets and that were not converging to a clear best-fit value in our numerical optimization (which was carried out including the ‘FindMinimum’ function in Mathematica 10.0 and higher^35^), indicating that super-diffusive behavior extends across all length scales in the dataset. Therefore, for these epidemics, in the final ML estimation procedure we set the factor $\exp \left( { - \frac{r}{\kappa }} \right)$ equal to 1 (corresponding to assuming $\kappa =\infty$) to remove the convergence problems. For the epidemics 1–5 (i.e. the epidemics without movement ban) the estimate of the scaling exponent $\alpha$ ranges between 0.66 to 1.83 (with the majority of values between 1.45 and 1.83) and for the epidemics 6–11 (with movement ban) it ranges between 2.36 and 2.68; i.e. we observe a perfect separation of the parameter ranges between epidemics with and without movement ban. The AIC and the AIC_0_ are significantly different (difference > 2) only for epidemics 4 and 5 (truncated Lévy-walk kernel performing better than the reference kernel). For the kernel estimation results taken from the literature (epidemics 1, 7 and 11), that literature gives results for non-truncated Lévy-flight kernels; i.e. no estimates for κ are available. The characteristic distance ${r}_{0}$ ranges between 0.18 km and 2.7 km, i.e. spanning roughly one order of magnitude across the different epidemics. Differences in ${r}_{0}$ are immaterial to the long-distance shape of the kernel as the latter is fully determined by the parameters $\alpha$ and $\kappa$; we note that this is illustrated by Fig. S1 where the graphs of the kernels for the epidemics 1–3 ($\kappa =\infty$ and $\alpha$ between 1.45 and 1.83) have very similar long-distance shapes and the same is observed for those of epidemics 6–11 ($\kappa =\infty$ and $\alpha$ between 2.36 and 2.68), and the graphs for the kernels of epidemics 4 and 5 each have a long-distance shape different from all other graphs due to a unique and finite value for $\kappa$. As the kernel amplitude λ_0_ does not inform about the distance dependence of transmission, we only report its estimated values in Table S1 for completeness.

**Table 3:** Estimations for the 11 epidemics of the Lévy-walk kernel parameters *r*_0_, $\alpha$ and $\kappa$ (with confidence bounds between brackets), the corresponding AIC, the AIC_0_ for the reference kernel estimation, and the applied control strategy (in order of increasingly stringent measures: no movement ban nor zoning (NMNZ), no movement ban but zoning (NMZ), and a movement ban (M)). For the kernel parameters of the transmission kernel for FMD in Japan (epidemic 11) no confidence bounds were reported in^27^.

Nr	Strategy	r_0_ (km)	$\alpha$	$\kappa$(km)	AIC	AIC_0_
1	NMNZ	0.69 (0.41, 1.07)	1.72 (1.54,1.93)	N/A	N/A	N/A
2	NMNZ	0.36 (0.02,1.64)	1.83 (1.45,2.39)	∞	437.5	436.632
3	NMNZ	2.5 (0.48,11.1)	1.45 (1.1,2.1)	∞	699.47	698.566
4	NMZ	1.32 (0.28,3.44)	1.70 (1.39,2.09)	173 (90,932)	24,125.3	24,130.8
5	NMZ	0.18 (0,18.7)	0.66 (0.38,2.13)	25.3 (20.3,45.9)	16,767.9	16,795.5
6	M	0.64 (0.43,0.96)	2.56 (2.36,2.82)	∞	6800.09	6801.96
7	M	1.33 (1.1, 1.56)	2.68 (2.58,2.78)	N/A	N/A	N/A
8	M	0.76 (0.17,2.64)	2.36 (1.86,3.21)	∞	505.884	505.015
9	M	2.7 (1.2,6.1)	2.53 (2.03,3.46)	∞	3089.80	3090.99
10	M	0.78 (0.003,4.05)	2.47 (1.75,3.98)	∞	206.852	205.017
11	M	0.58	2.47	N/A	2093.08	2094.32

**Figure 1:**
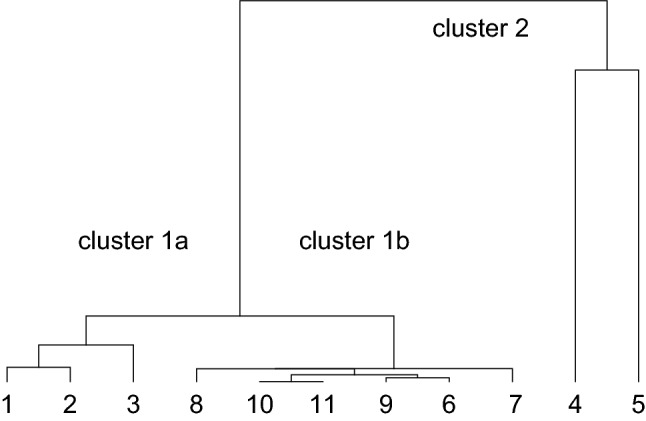
Dendrogram of the hierarchical clustering of the value pairs $\left\{\alpha ,\kappa \right\}$ for the 11 epidemics; two main clusters 1 and 2 are indicated, and within cluster 1 the two (sub)clusters 1a and 1b could be distinguished.

In Fig. 1 we show the dendrogram result from the hierarchical clustering analysis of the value pairs $\left\{\alpha ,\kappa \right\}$ for the all 11 epidemics. This dendrogram indicates two main clusters, labelled 1 and 2, in which cluster 1 could be subdivided in two sub-clusters 1a and 1b. The clusters coincide completely with the grouping according to strategy (p < 0.001): The epidemics 1–3 with strategy NMNZ are all located in cluster 1a, the epidemics 6–11 with strategy M are all located in cluster 1b, and the epidemics 4 and 5 with strategy NMZ are both located in cluster 2.

## Discussion

We gathered transmission kernel parameter estimates for 11 livestock disease epidemic datasets that together cover a broad range of host–pathogen combinations as well as combinations of control measures applied. A subsequent hierarchical clustering analysis of the parameter value combinations showed a maximal correlation between clusters and a grouping according to control measures. In particular, we observed a full separation of the ranges of estimates for the scaling exponent $\alpha$ between strategies with movement ban (epidemics 6–11, coinciding with cluster 1b, where $\alpha$ ranges between 2.36 and 2.68) and without movement ban (epidemics 1–5, coinciding with clusters 1a + 2, where $\alpha$ ranges between 0.66 and 1.83). We observed that for the strategies without movement bans the scaling exponent is most often close to (and never significantly different from) the universal value of approximately 1.6 identified in analyses of human travel mobility patterns. This suggests that in absence of movement bans, the distance dependence of between-farm transmission is strongly determined by the pattern of between-farm animal transports; and that this pattern is characterized by a scaling exponent taking a value close to the universal value for human mobility. Indeed, modelling suggests that the power-law scaling of movements of infected individuals would be directly reflected in the spatial transmission pattern^36^. In line with this interpretation, we observe that only for the two epidemics with zoning measure (NMZ), the inclusion of the cutoff parameter $\kappa$ leads to a better fitting model, suggesting that the zoning measure restricts the universal power-law dependence to distances in the order of the size of the zone. The applied protection zone of the 2006 BT epidemic in Germany (epidemic 11) was about 20 km and if zones overlapped they were merged to one larger zone^11^. The estimate for the cutoff distance $\kappa$ epidemic 5 is 25.3 (20.3,45.9) km which we deem fully consistent with these zoning measures. The estimate for the cutoff distance in the 2006 BT epidemic in Belgium (epidemic 4) is 173 (90,932) km; as in this analysis (in contrast to the previous analysis reported in^11^) the populations of the Netherlands and France were also included, this 173 km represents an informative distance. This estimate is consistent with Belgium being declared one protection zone with the ‘radius’ of the country being about 140 km. The close correspondence, in both cases 4 and 5, between zoning measure and kernel shape further underpins the interpretation that constraining animal transports to within defined zones limits the dominant transmission route to distances within the extent of the zone. We note that although the analyses of the epidemics 1–3 and 6–11 do no produce a finite cutoff parameter, this is still consistent with the expectation that imposed export bans did produce a zoning effect on the scale of the country in question, as such an effect is not identifiable in an analysis including only the farm population within that country. Although the truncated Lévy-walk kernel is performing only better for epidemics 4 and 5 and similar for the other epidemics, the use of the truncated Lévy-walk kernel enables the comparison of the parameters of the different transmission kernels yielding further insight*.*

As noted above, for the six epidemics with movement bans the estimated scaling exponent ranges between 2.36 and 2.68 which is clearly different from the range (0.66–1.83) estimated for the 5 epidemics without movement ban, the difference being of the order of 1.0. The fact that the imposition of a movement ban produces such a dramatic shift of α, implies that in absence of a movement ban, the longer-distance disease transmission is mainly driven by animal transports. Other possible transmission routes, such as human and fomite movements unrelated to animal transports, movements of wild animals and wind-borne virus dispersal, together may only play a minor role in the longer-distance disease transmission as long as no movement ban is in place. After imposition of a movement ban these other possible transmission routes are the ones remaining, and are yielding a scaling exponent that, compared to the situation without movement ban, seems to be increased by an amount of the order of 1.0. This difference of about 1.0 can be interpreted if we make two assumptions. First, we assume that the dispersal or movements underlying the remaining transmission routes are occurring predominantly in random directions, i.e. unlike animal transports these are not necessarily directed towards a neighboring farm. Second, we assume that these dispersal or movement processes are also described by a scaling exponent of approximately 1.6. The difference of about 1.0, which corresponds to a factor 1/r, can then be explained from taking into account that for transmission to occur, the random movement direction of the infectious material has to match the direction towards a specific susceptible farm; the probability that this match occurs is inversely proportional to the distance r to the susceptible farm^36^. Regarding the first assumption, we note that the indirect between-farm contacts occurring when e.g. feed delivery or egg collection trucks visit multiple farms on the same day, are clearly directed to the farms as destinations. I.e. these types of contacts do not correspond to movement with a random direction. This means that our assumption corresponds to a situation where only a minor part of infections can be attributed to these types of contacts, as was the case for epidemic number 9^37^. Regarding the second assumption, transmission via human movement, e.g. the route of a passerby accidentally connecting two farms, fits well into this interpretation as, Lévy-walk patterns with a scaling exponent of 1.6 have been identified for human movement^23^. This interpretation would thus support the hypothesis that ‘random’ human movement is the most important between-farm transmission route once animal movement bans are in place. However, the value of 1.6 does not exclusively point towards human movement. Concerning movement of wild animals, it is known of some species that they also move according a Lévy-walk pattern with a scaling exponent of about 1.6^38^. Concerning wind, specific atmospheric conditions can lead to aerial dispersal following a Lévy-walk pattern with a scaling exponent of about 1.5^39^. In addition, we note that our interpretation also implicitly assumes that the movement of infectious material between farms is fast enough such that pathogen survival is not influencing the scaling of transmission with distance.

Our results show that after imposing a movement ban, transmission retains its super-diffusive character, although the movement ban does reduce the longer-distance transmission risks by increasing the scaling exponent α from around 1.6 to around 2.6. As long as transmission has a super-diffusive character (power-law dependence on distance), it will be difficult to spatially confine it using local control measures such as ring culling or ring vaccination.

The analyses in this paper provide a conceptual framework for analysis of spatial livestock disease spread and control. In addition to its relevance for interpreting past epidemic patterns and underpinning model extrapolations from these patterns, our approach provides a framework for assessing the spatial transmission of emerging livestock diseases, i.e. diseases for which no previous epidemic is available to estimate kernel parameters. As a prior model, the between-farm transmission could be assumed to follow a Lévy-walk kernel with parameter values as follows. The exponential power $\alpha$ would be assumed to be about 2.6 if a movement ban is put in place and 1.6 without a movement ban. The spatial limitation $\kappa$ would be assumed to equal the radius of the protection zone in case no movement ban is implemented and the radius of (a circular area approximating) the country when import bans are imposed by neighboring countries. According to Table 3, the characteristic distance r_0_ seems to be of the order of 1 km across all epidemics included. The remaining information needed for the Lévy-walk kernel, namely the value of the (relative) amplitude of the between-farm transmission (λ_0_) for the emerging disease in question, could be treated as a scenario parameter, to be varied within a plausible range. The resulting prior model can be used for analyses that can comprise risk maps for the spread of the emerging disease as well as more detailed evaluation of control measures by means of kernel model simulations^6,9^.

## Supplementary Information

Supplementary Information.

## Data Availability

All data generated and analyzed during this study are included in this published article and its Supplementary Information file.
