# Supplementary material for: Common features in spatial livestock disease transmission parameters
Source: Sci Rep. 2023 Mar 2;13:3550. doi: 10.1038/s41598-023-30230-w (PMC9981765; doi:10.1038/s41598-023-30230-w)
Supplement: Supplementary file 1 — Supplementary Information. [file 41598_2023_30230_MOESM1_ESM.pdf]

**Table S1** The re-estimated Lévy-walk kernel parameter $\lambda_0$ (with confidence bounds between brackets) for the 8 epidemics in Table 1 with kernel model of existing analysis 'reference'.

| Nr | $\lambda_0$ |
|---|---|
| 2 | 0.0066 (0.0013,0.3) |
| 3 | 0.285116 (0.0921064,0.972282) |
| 4 | 0.000062 (0.000028,0.000266) |
| 5 | 0.00013862 (0.00002,∞) |
| 6 | 0.0073 (0.0047,0.0115) |
| 8 | 0.0044 (0.0011,0.025) |
| **9** | 0.0036 (0.002,0.0071) |
| 10 | 0.015 (0.0024,37) |

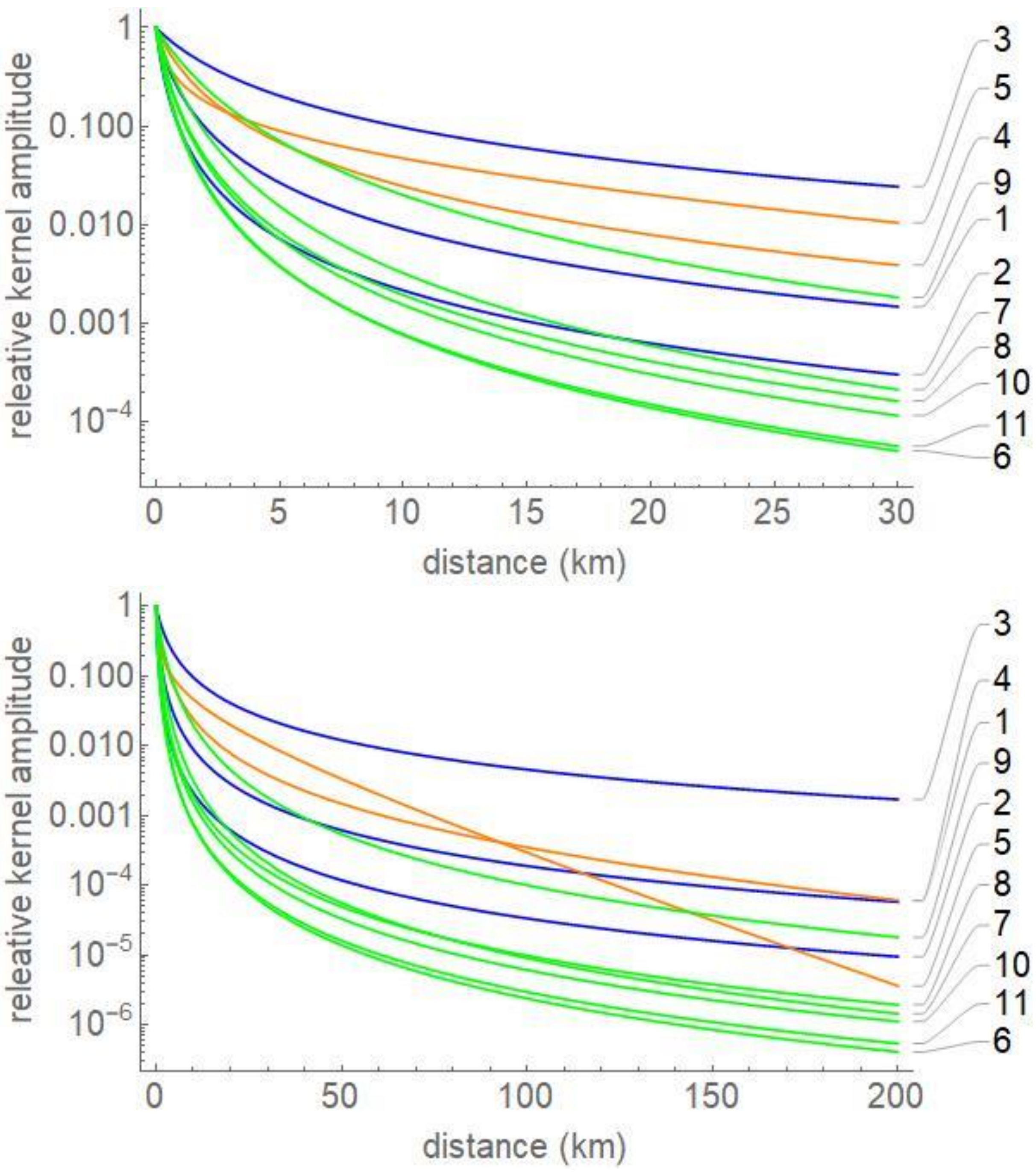


**Figure S1** Transmission kernels labelled with the epidemic dataset number for the control measure 'no movement ban nor zoning' (NMNZ) (blue), 'no movement ban but zoning' (NMZ) (orange), and 'a movement ban' (M) (green); the amplitude $\lambda_0$ of the transmission kernels is normalized to 1 and the kernels are plotted on two different distance scales.
